# Role of c-ABL in DENV-2 Infection and Actin Remodeling in Vero Cells

**DOI:** 10.3390/ijms26094206

**Published:** 2025-04-29

**Authors:** Grace Paola Carreño-Flórez, Alexandra Milena Cuartas-López, Ryan L. Boudreau, Miguel Vicente-Manzanares, Juan Carlos Gallego-Gómez

**Affiliations:** 1 Translational Medicine Group—School of Medicine, Universidad de Antioquia, Medellín 050010, Colombia; gcarrenoflorez@gmail.com (G.P.C.-F.); alexandra.cuartas@udea.edu.co (A.M.C.-L.); 2 Department of Internal Medicine and Abboud Cardiovascular Research Center, Carver College of Medicine, University of Iowa, Iowa City, IA 52242, USA; ryan-boudreau@uiowa.edu; 3 Molecular Mechanisms Program, Centro de Investigación del Cáncer, Instituto de Biología Molecular y Celular del Cáncer, Consejo Superior de Investigaciones Científicas (CSIC)-University of Salamanca, 37007 Salamanca, Spain

**Keywords:** DENV, c-ABL, cellular Abelson Tyr kinase, actin, imatinib, artificial microRNA

## Abstract

In this study, we address the role of c-ABL (cellular Abelson Tyr kinase) in the cytoskeletal rearrangements induced by DENV (Dengue virus) infection in mammalian cells. Using the specific inhibitor imatinib and targeted RNA interference, we show that c-ABL is necessary for viral entry and subsequent ENV (DENV envelope protein) accumulation in infected cells. In addition, c-ABL targeting attenuates F-actin reorganization induced by DENV infection. Together with the involvement of c-ABL in endothelial dysfunction induced by DENV and host secreted factors, our findings strongly suggest that c-ABL is a potential host-targeted antiviral that could control DENV infection and/or its evolution to more severe forms of the disease.

## 1. Introduction

Dengue is one of the most common mosquito-borne viral diseases worldwide [1,2]. Its causal agent is Dengue virus (DENV), a flavivirus related to Zika (ZIKV), yellow fever (YFV), and Chikungunya (CHIKV) viruses. Most flaviviruses belong to the arbovirus family, short for arthropod-borne viruses, and are transmitted to humans and other vertebrates through the bites of blood-feeding arthropods, such as mosquitoes and ticks [3]. DENV constitutes a major threat to global health [4]. Over one billion people in tropical and subtropical areas are potentially exposed to the disease. Climate change and overpopulation are two major causes of the current outbreak of dengue in the Americas, which is the largest to date (https://www.cdc.gov/dengue/outbreaks/2024/index.html, accessed on 22 January 2025).

DENV is an RNA-based virus and a core member of the *Flaviviridae* family. Its genome encodes a large, single polyprotein that is proteolyzed inside host cells to produce three structural proteins (capsid, C; pre-membrane, prM; envelope, ENV) and seven non-structural (NS) proteins, NS1, NS2A, NS2B, NS3, NS4A, NS4B and NS5 [5,6]. Four major serotypes of DENV (1–4) have been identified [7]. Subtle differences among serotypes stem from the lack of proofreading activity of NS5 and natural selection of positive mutations [8].

Most Dengue cases are mild, with modest fever and joint pain and swelling. However, selected patients develop an aggressive form of the disease termed severe Dengue. This form is characterized by violent hemorrhagic crises that can only be treated with palliative care [1]. The etiopathology of severe Dengue is poorly characterized, but recent advances have revealed a crucial role for non-neutralizing antibodies and endothelial dysfunction. Antibodies generated during an initial round of infection neutralize additional infections triggered by the same serotype. However, a different DENV serotype or a mutated form of the same variant may not be neutralized by the antibodies produced during the initial infection. These non-neutralizing antibodies may instead promote viral entry in Fc-bearing cells, such as macrophages. This process is termed ADE (Antibody-Dependent Enhancement of infection), and it increases viral burden and systemic inflammation, which may lead to generalized hemorrhage [9]. This form of the disease displays a manifold increase in mortality and morbidity compared to regular Dengue. Interestingly, severe Dengue is mostly observed in the post-febrile phase of the disease [10]. This strongly suggests that systemic inflammation, which is often observed during the febrile phase, may not be the only factor involved in the onset of severe Dengue. In this regard, we have recently reported that DENV infection triggers trans-differentiation events in endothelial cells that may compromise their barrier function [11,12].

Efforts to develop an efficient Dengue vaccine have been hampered by the variability of the virus. In 2018, the European Medicines Agency (EMA) approved the use of a vaccine against DENV (DengVaxia^®^) [13], but clinical evidence indicates that it only confers protection in patients pre-exposed to the virus. The vaccine actually increases the risk of severe Dengue in naïve patients (probably due to ADE, as the vaccine likely boosts the production of non-neutralizing antibodies) [14]. This has shifted the focus of the research towards direct antivirals (DA) and host-targeted antivirals (HTA). DAs target viral elements (RNA polymerase, structural proteins, etc.) to prevent replication and/or infection. Conversely, HTAs are designed against endogenous cellular machinery that is essential for the completion of the viral cycle. HTAs have shown promising results against host cell targets involved in the cycle of a broad range of viruses, including DENV [15,16], Hepatitis C virus (HCV) [17], West Nile virus (WNV) [15], and SARS-CoV-2 [18,19,20]. HTAs can target very different pathways. For example, viruses hijack molecular motors associated with microtubules or microfilaments to promote nuclear localization and/or exocytosis of assembled virions [21]. A recent example of HTA is the targeting of ACE2 to prevent SARS-CoV-2 infection [22].

c-ABL (cellular Abelson Tyr kinase) proteins have previously been described as signaling intermediates involved in DENV infection and spreading. There are two isoforms, c-ABL1 and c-ABL2, encoded by the *ABL1* and *ABL2* genes, respectively. c-ABL1 is well-known for its oncogenic capability when truncated and fused to BCR (breakpoint cluster region protein). The resulting BCR-ABL1 fusion protein displays constitutive kinase activity, driving unchecked cell growth in several types of leukemia, such as Chronic Myeloid Leukemia (CML) and Acute Lymphoblastic Leukemia (ALL) [23]. In a non-oncogenic context, c-ABL lies downstream of growth factor receptors and integrins, controlling multiple signaling pathways that include cell proliferation and survival, cytoskeletal remodeling, and apoptosis [24]. Some of these effects take place in the cytoplasm, whereas others rely on the nuclear translocation of c-ABL, which is enabled by three tandem Nuclear Localization Sequences (NLS) localized in the center of the molecule [24]. In resting cells, the kinase activity of c-ABL remains off because of the interaction of the central, (Src-Homology) SH3-SH2-containing domain with the Tyr kinase catalytic site, which inhibits substrate and ATP binding [25]. Interaction with SH2 ligands (such as phosphorylated Tyr residues in other cellular proteins) and the phosphorylation of c-ABL itself hampers the interaction of the SH3-SH2 loop with the catalytic site, relieving its inhibition and enabling the phosphorylation of downstream targets. Importantly, c-ABL contains an Actin-Binding Domain (ABD) at the C-terminus [26], which positions c-ABL as an important regulator of actin dynamics [24].

c-ABL is important for the pathogenic cycle of various bacteria and viruses. It is involved in the replication of viruses such as Ebola and Hepatitis virus B and C (HBV and HCV) [27,28,29]. c-ABL also mediates the host’s response to infection, e.g., during epithelial barrier disruption [30,31,32] and the antiviral immune response [33]. c-ABL is also involved in the reorganization of the host’s cellular cytoskeleton and intracellular traffic during infection [34,35,36,37]. It can also interact directly with viral proteins [27,38]. For example, c-ABL regulates the attachment of polyomavirus, entry, and genome release of Human Immunodeficiency Virus (HIV)-1 and coxsackievirus [36,37,39] and actin comet formation and virion budding in Vaccinia infection [34,35]. Whereas c-ABL seems involved in DENV post-entry events [40], its specific function in DENV pathogenesis remains mostly unknown.

Increasing evidence shows that infection by DENV virus remodels actin filaments, microtubules and vimentin to promote viral entry, replication and budding [41,42,43,44,45,46]. Particularly, actin dynamics is critical for viral entry, synthesis of viral structural proteins, and viral release [45,46]. Here, we show that either RNAi-mediated silencing or pharmacological inhibition of c-ABL during DENV infection in interferon (IFN)-I-deficient Vero cells leads to accumulation of the envelope protein (ENV) at the intracellular level through a mechanism involving actin remodeling. These findings shed light on key cellular mechanisms for DENV infection that could be used to reposition existing drugs as HTA.

## 2. Results

### 2.1. Efficient Depletion of c-ABL Using miRNA

**Figure 1:**
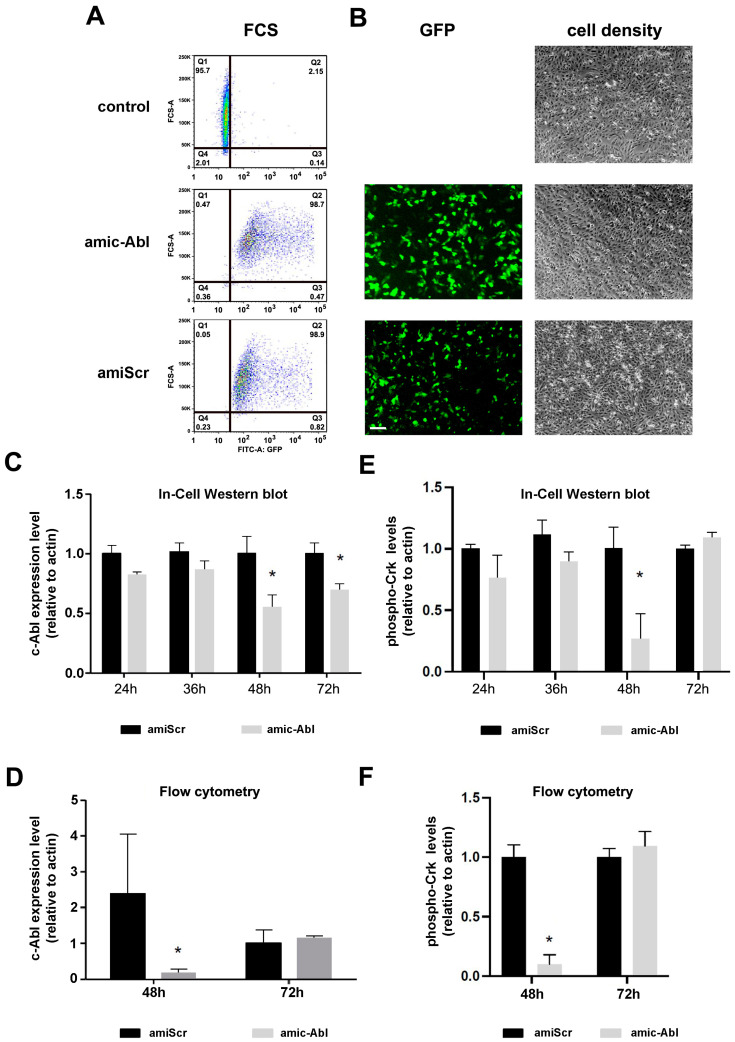
Artificial miRNA induces silencing of c-ABL and phospho-Crk inhibition. (**A**) Flow cytometry profiles of Vero cells transfected for 48 h with pBluescript (control) and amiRNA targeting c-ABL or a scrambled form (see Section 4 for details). (**B**) Representative fluorescent images of cells as in (**A**). Scale bar = 50 µm. (**C**,**D**). Kinetics of c-ABL expression by In-Cell Western blot (**C**) and flow cytometry (**D**). By ICW, c-ABL levels were measured 24 h, 36 h, 48 h, or 72 h post-transfection. Actin levels were also estimated by ICW and used for relativization. By FCS, levels were evaluated at 48 and 72 h post-transfection. Cells were co-stained with fluorescent phalloidin to obtain a relativized ratio. (**E**,**F**) Levels of phospho-Crk were estimated by ICW (**E**) or flow cytometry (**F**) as in (**C**,**D**). Data represents the mean ± SEM relative to control from three independent experiments. *p*-values were determined by two-tailed unpaired *t*-test (*: *p* < 0.05).

To deplete c-ABL expression and test the role of this kinase in DENV infection, we first developed a vector containing an artificial miRNA that targets c-ABL1 [47]. This vector co-expresses GFP, which we used to determine the efficiency of transfection by flow cytometry. Flow cytometry graphs show a very high transfection efficiency, over 95%, compared to non-transfected control cells (Figure 1A). We also included a control plasmid containing a scrambled sequence that does not target any specific mammalian gene (bottom graph, Figure 1A). The inclusion of the cABL-targeting artificial miRNA sequence did not affect transfection efficiency. Similar results were observed when cells were examined in a fluorescence microscope (Figure 1B).

We next addressed the efficiency of c-ABL depletion by In-Cell Western blot (Figure 1C) and flow cytometry (Figure 1D). At 48 h, we observed reproducible depletion of c-ABL by both techniques. At 72 h, we observed c-ABL recovery, possibly due to the degradation of the vector, as suggested by GFP fluorescent decay, or another alternative compensatory mechanism. To assess the functional outcome of c-ABL depletion, we also measured the levels of phosphorylation of Crk-II in Tyr221, a well-known target of c-ABL [48]. We observed a significant reduction in the levels of phospho-Crk-II at 48 h by ICW and flow cytometry that returned to baseline at 72 h (Figure 1E,F), consistent with the depletion kinetics of c-ABL.

Together, these results demonstrate the efficiency of our vector-based artificial miRNA to specifically target c-ABL expression in Vero cells.

### 2.2. c-ABL Targeting with Imatinib or miRNA-Based Depletion Triggers DENV-2 Envelope Protein Accumulation and Actin Reorganization in Vero Cells with Different Kinetics

**Figure 2:**
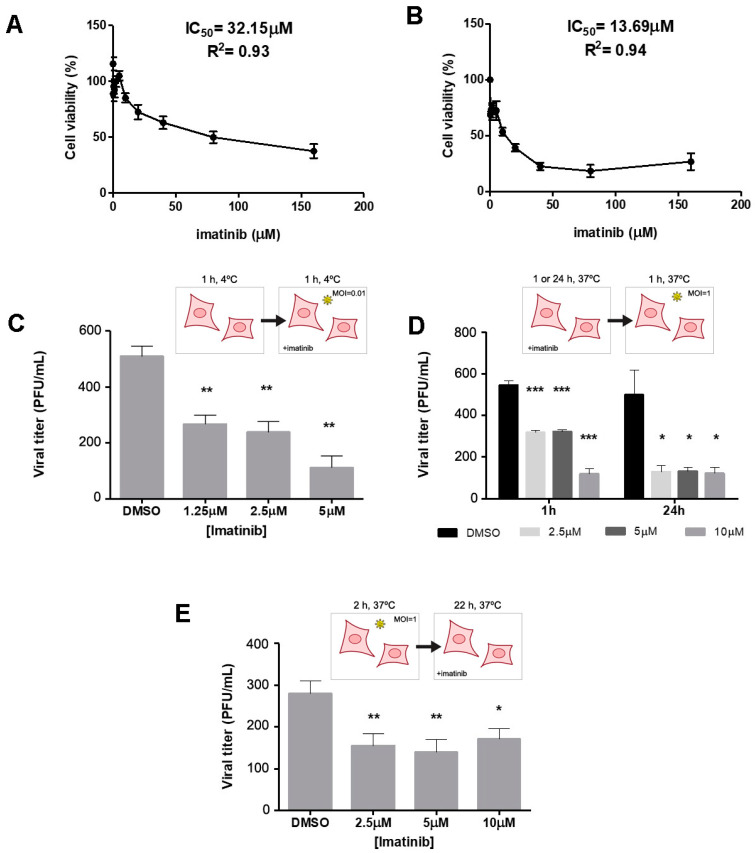
c-ABL targeting reduces DENV-2 infection during binding, entry, and post-entry events and promotes ENV protein accumulation (**A**,**B**). Vero cells were treated with the indicated doses of imatinib for 24 h (**A**) and six days (**B**). Data are the mean ± SD of the toxicity of imatinib. Bars represent three biological experiments done in triplicate. (**C**) DENV at an MOI of 0.01 was added to pre-chilled (4 °C, 1 h) Vero cells. Cells were incubated for 1 h at 4 °C, medium was removed, and the PFU assay was carried out as indicated in Section 4. (**D**) Vero cells were preincubated at 37 °C with three different imatinib concentrations. After that, the inhibitor was removed, DENV added at an MOI of 1, and samples collected at 1 h and 24 h post-infection. (**E**) Vero cells were infected with DENV at an MOI of 1 for 2 h at 37 °C. Excess virus was removed, and cells were incubated for 22 h in fresh medium with the indicated concentrations of imatinib. For (**A**–**E**), data represent the mean ± SD of three independent experiments performed in triplicate. *p*-values were determined by two-tailed unpaired Student’s *t*-test (*: *p* < 0.05, **: *p* < 0.01, *** *p* < 0.001).

To study the effect of c-ABL targeting in DENV infection, we also used imatinib, a well-characterized c-ABL inhibitor used to treat BCR-ABL-positive leukemia [49]. Initially, we assessed the ability of imatinib to induce cytotoxicity using the MTT assay [50]. We found that, consistent with previous reports [51], imatinib triggered cytotoxicity in the micromolar range, assessed 24 h (Figure 2A) and six days (Figure 2B) post-addition. We next investigated the effect of imatinib on the binding, entry, and expansion of DENV in Vero cells. First, we incubated imatinib-treated Vero cells at an MOI of 0.01 at 4 °C to measure virus binding. We found that imatinib interfered with DENV binding to the surface of the cells (Figure 2C). We next examined the ability of imatinib to interfere with DENV entry into target cells. Cells were pre-treated with imatinib and infected in the absence of the drug. We found that imatinib also interfered with DENV entry (1 h post-infection, Figure 2D). Importantly, inhibition was achieved with a lower dose at a longer (24 h) incubation period, suggesting that c-ABL inhibition is long-lasting. Finally, we assessed the role of c-ABL in post-entry events by infecting the cells in the absence of imatinib, then adding imatinib once excess virus had been removed (Figure 2E). We found that this protocol significantly decreased the PFU at all imatinib doses, indicating a role for c-ABL in post-entry events.

**Figure 3:**
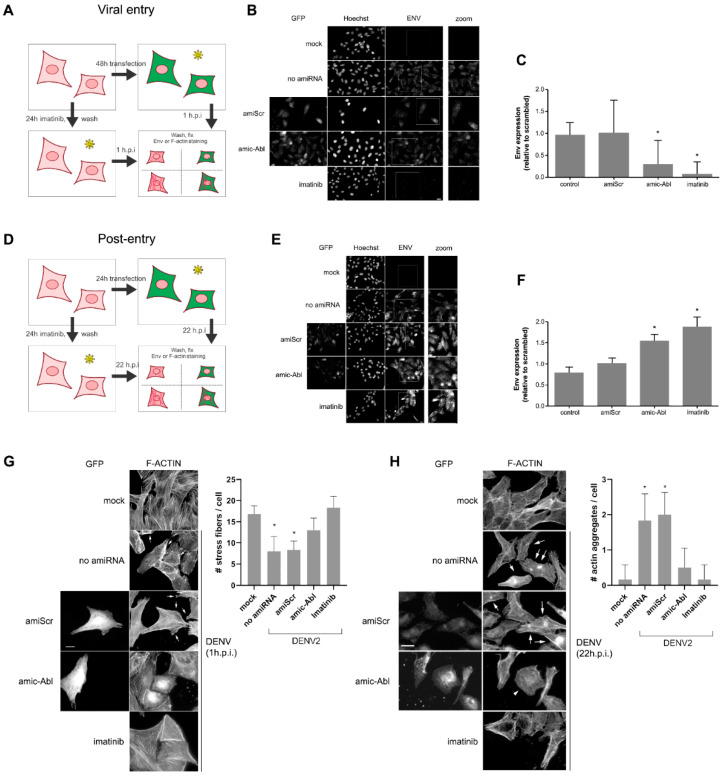
c-ABL targeting alters actin organization during late infection events. Vero cells transfected as indicated and infected 24 or 48 h post-transfection with DENV at an MOI of 1 for 1 h at 37 °C. Cells infected 48 h post-transfection were fixed 1 h post-infection to measure virus entry-related events (**A**–**C**), and cells infected 24 h post-transfection were fixed after 22 h to measure post-virus entry events (**D**–**F**). Where indicated, cells were treated with imatinib as in Figure 2. Scale bar = 20 µm. In (**B**,**D**), each bar represents the mean ± SEM relative to scrambled control from three independent experiments. *p*-values were determined by two-tailed unpaired Student’s *t*-test (*, *p* < 0.05). (**E**,**F**) Vero cells transfected as indicated were infected 24 h post-transfection with an MOI of 1. After 1 h (**G**) and 22 h (**H**) post-infection (h.p.i.), cells were fixed, and F-actin was visualized using fluorescent phalloidin. In (**G**), arrows point to actin ruffles in the cell periphery. Graph insert depicts stress fiber quantification as indicated in Section 4. *n* = 30 from two independent experiments. In (**H**), arrows point to actin aggregates, and arrowhead indicates a round, c-ABL-depleted cell. Graph insert depicts actin aggregate quantification as indicated in Section 4. *n* = 30 from two independent experiments. *p*-values were determined by two-tailed unpaired Student’s *t*-test (*, *p* < 0.05). Scale bars = 20 µm.

We next examined the accumulation of ENV protein in cells in which we had silenced c-ABL1 (Figure 3A,D). To do this, we visualized ENV using fluorescence microscopy in cells transfected with the artificial miRNA described in Figure 1. We observed a significant decrease in the levels of ENV in cells depleted of c-ABL shortly (1 h) after infection (Figure 3B,C), indicating that cells depleted of c-ABL display decreased infection susceptibility. Conversely, after 22 h post-infection, we detected no significant difference in the levels of intracellular ENV in cells depleted of c-ABL or treated with imatinib compared to control cells (Figure 3E,F). This strongly suggests that c-ABL is involved in the early accumulation of ENV during viral entry.

Finally, we addressed the effect of c-ABL depletion in F-actin remodeling induced by DENV infection. When Vero cells were infected with DENV-2 (1 h.p.i.), we observed a reduction of stress fibers and increased ruffling (indicated with arrows) in infected cells, which was not as prominent in c-ABL-depleted cells (Figure 3G). At late stages of DENV-2 infection (22 h.p.i.), control cells displayed well-organized F-actin stress and also disorganized F-actin clusters (arrows), whereas c-ABL-depleted cells (arrowhead) displayed fewer filaments, a round phenotype, and the absence of clusters (Figure 3H). Imatinib had a stronger but comparable effect to the c-ABL-targeting microRNA. The effects observed when c-ABL was depleted or inhibited are comparable to the phenotype observed in c-ABL knock-out MEFs [52]. These results indicate that c-ABL is required for actin remodeling during DENV-2 infection, specifically actin ruffling during infection and stress fiber reorganization and actin aggregation at later time points.

## 3. Discussion

The data herein supports the possibility of using c-ABL as a target against DENV infection, thus suggesting that imatinib, a specific c-ABL inhibitor used in therapy for BCR-ABL-positive leukemia, could be repositioned as a host-targeted antiviral (HTA) to treat Dengue. This could be particularly useful to treat severe Dengue, which, despite being a rare occurrence, bears a high mortality rate and has no specific treatment. Here, we show that c-ABL depletion decreases DENV binding and ENV accumulation during viral entry and also decreases actin remodeling in response to infection. This suggests that c-ABL potentially controls DENV infection at multiple levels, including entry, post-entry, and late events in the viral cycle, possibly hijacking microfilaments, as described before [34].

At the entry level, c-ABL could control the levels, or dynamics, of potential DENV receptors. DENV seems capable of using multiple different receptors, including GAGs (glycosaminoglycans, mannose receptors, C-lectin type receptor DC-SIGN (Dendritic Cell-specific ICAM-3-grabbing nonintegrin), and even Fc receptors [53]. c-ABL mediates signaling through multiple membrane receptors, e.g., integrins, and its function underneath the plasma membrane makes this an attractive possibility. The fact that c-ABL is an important mediator of actin remodeling underlines the possibility that it may be controlling DENV entry by altering the availability or internalization of potential DENV receptors [54].

The DENV post-entry involvement of c-ABL is underscored by the reduced accumulation of ENV. DENV disassembly occurs after internalization, and ENV undergoes multiple conformational changes that lead to deployment of the viral nucleocapsid in the cytosolic space [55]. It is possible that c-ABL depletion inhibits these changes or generally interferes with the viral morphogenesis events that are controlled by cytoskeletal elements [21]. It is also possible that it accelerates ENV degradation. Conversely, c-ABL does not seem to participate in de novo ENV synthesis, as seen by the similar, or even higher, levels of ENV in c-ABL-depleted or -inhibited cells after 24 h. At this time point, the higher levels of ENV correspond to newly synthesized protein, part of which is incorporated into assembling virions.

Finally, c-ABL also mediates cytoskeletal rearrangements observed during the late stages of the viral cycle. c-ABL mediates actin organization, partly due to its actin-binding ability [24]. Diverse reports have indicated that c-ABL inhibition induces stress fiber formation. We also observe this effect when the cells are incubated with imatinib, while it is less obvious when c-ABL is depleted using RNAi. However, c-ABL depletion was sufficient to prevent DENV-promoted membrane ruffling. Importantly, infection also promotes the formation of actin aggregates, which were not as prominent in c-ABL-inhibited cells. While c-ABL may control other mandatory steps of DENV replication or assembly, our data support that the inhibitory effect of c-ABL targeting is due, at least in part, to its effect on actin remodeling. Since DENV-2 infection induces actin remodeling [45,56,57], our data support the notion that DENV-induced F-actin remodeling requires c-ABL, in agreement with previous results [40].

We propose a model in which c-ABL not only mediates early cytoskeletal rearrangements induced by DENV, e.g., actin ruffling, but also promotes the accumulation of F-actin in aggregates as cells increase their viral burden. In this sense, we can speculate that imatinib could be used to control cytopathic effects (including but not limited to F-actin aggregation) of the virus at later stages, opening the door to the exploration of imatinib as a repositioned agent to treat severe Dengue. This could also include other c-ABL inhibitors such as nilotinib, bosutinib, or dasatinib. In this regard, previous research has shown that dasatinib increases endothelial permeability through a ROCK-dependent mechanism [58]. Also, we cannot rule out other non-cytoskeletal effects of c-ABL, such as the regulation of transcriptional activation and phosphorylation of non-cytoskeletal targets.

It is important to note that the present study focuses solely on Vero cells, which do not develop IFN-I-based antiviral responses [59]. This facilitates viral infection, eliminating the effects due to this innate anti-viral response. However, similar observations have been conducted in human endothelial HMEC1 cells, which are not IFN-I-deficient [12].

The current non-excluding models of severe Dengue include ADE and endothelial dysfunction. ADE is triggered by antibody-dependent internalization, which in turn crucially depends on actin-based rearrangements [60]. Thus, a possibility is that c-ABL inhibition could decrease the impact of ADE towards severe Dengue. Also, we have shown that c-ABL inhibition decreases the onset of expression of mesenchymal markers upon DENV infection [12], suggesting that c-ABL decrease could also “normalize” the endothelium, decreasing hemorrhage during severe Dengue.

In summary, our data clearly show that c-ABL can be successfully employed to inhibit DENV infection at various levels, opening an avenue that could lead to the repositioning of imatinib as a potential HTA to treat severe Dengue.

## 4. Materials and Methods

### 4.1. Chemicals and Antibodies

Imatinib mesylate inhibitor was purchased from Selleck Chemicals (Houston, TX, USA). It was dissolved in DMSO at 1 mM and stored as a stock solution at −20 °C. Primary anti-Envelope antibody (α-ENV, clone GT214, mouse) and anti-phospho-CrkII (rabbit pAb) were purchased from Merck Millipore (St. Louis, MO, USA). Primary anti-c-ABL antibody was purchased from Santa Cruz Biotechnology (Santa Cruz, CA, USA). Anti-β-actin mouse antibody used for normalization was from LI-COR Biosciences (Lincoln, NE, USA). Anti-mouse- or anti-rabbit antibodies coupled with IRDye 680LT or IRDye 800CW were from LI-COR Biosciences. AlexFluor594-conjugated goat anti-mouse, goat anti-rabbit antibodies, and phalloidin and Hoechst 33258 were purchased from Thermo Invitrogen (Carlsbad, CA, USA). L-15 medium and Dulbecco’s Modified Eagle’s Medium (DMEM) were supplied by Merck. Fetal bovine serum (FBS) and tissue culture antibiotic cocktail Penicillin/Streptomycin (PS) were from Invitrogen.

### 4.2. Cell Lines and Virus

Dengue virus serotype 2 New Guinea strain (DENV-2 NGS) was provided by María Elena Peñaranda and Eva Harris (Sustainable Sciences Institute and the University of California, Berkeley, CA, USA). Vero cells (ATCC CCL-81) were maintained at 37 °C under 5% CO_2_. They were cultured in DMEM supplemented with penicillin (100 U/mL)-streptomycin (100 ug/mL). For routine passage, medium was supplemented with 10% FBS. For infection, serum was reduced to 2%. C6/36 HT cells were grown in L-15 medium supplemented with 10% FBS and penicillin (100 U/mL)-streptomycin (100 ug/mL) at 34 °C and used to amplify DENV-2 NGS. For viral propagation, C6/36 HT cells were infected at MOI of 0.01 for seven days. Supernatants containing the viruses were collected and frozen at -80 °C, and the PFUs (Plaque-forming units) were quantified by plaque assay, as briefly described in our recent study [56].

### 4.3. Plasmids, Transfection, and RNAi

*ABL1* gene silencing was achieved using plasmid-based artificial microRNA (miRNA) expression vectors that co-express a GFP reporter [61]. Specifically, artificial miRNAs harboring small interfering RNAs targeting *ABL1* (miABL1) or scrambled control sequence (miScrambled) were cloned into the expression plasmid pFBAAVmU6mcs-CMVeGFP SV40pA, which is available from the Viral Vector Core at Carver College of Medicine (University of Iowa, Iowa City, IA, USA). Plasmid transfections were carried out using Lipofectamine 2000 (Thermo Fisher Scientific, Waltham, MA, USA) following the manufacturer’s instructions.

The transfection efficiency of the plasmids pFBAAVmU6miABL1-CMVeGFP SV40pA (mi*ABL1*) and pFBAAVmU6miScramble-CMVeGFPSV40pA (scrambled version) was confirmed through the expression of the GFP reporter [61,62].

### 4.4. Imatinib Cytotoxicity and Inhibition Treatments

Cytotoxicity was assessed using the MTT (3-(4,5-Dimethylthiazol-2-yl)-2,5-Diphenyltetrazolium bromide) assay [50]. IC_50_ was determined in Vero cells following a previously standardized protocol for DENV infection. Briefly, Vero cells were seeded at 80% confluence in 96-well plates and incubated with the indicated doses of imatinib for either 24 h or 6 days at 37 °C and 5% CO_2_. After each time point, the medium was replaced with 50 μL of MTT and incubated for 2.5 h at 37 °C. Isopropanol was added to dissolve the formazan crystals, and absorbance was measured in a benchtop spectrophotometer (Benchmark reader, Bio-Rad Laboratories, Hercules, CA, USA). IC_50_ values were obtained using a linear regression model.

### 4.5. Virus Binding, Entry, and Post-Entry Assays

To determine the effect of imatinib in the different steps of DENV-2 infection, Vero cells were seeded at 80% confluency in 24-well culture plates (2.5 × 10^4^ cells per well). For viral attachment, Vero cells were incubated at 4 °C for 1 h, followed by infection with DENV-2 (MOI of 0.01) in the presence of imatinib (1.25 μM to 10 μM) or without treatment (mock) for 1 h at 4 °C. Cells were then washed once with PBS and fixed. To evaluate viral entry, Vero cells were treated with imatinib (1.25 μM to 10 μM) for 1 h and 24 h at 37 °C and 5% CO_2_, followed by DENV-2 infection (MOI of 1). To evaluate post-entry steps, Vero cells were infected with DENV-2 (MOI of 1) for 2 h at 37 °C and 5% CO_2_, then incubated with medium containing imatinib (1.25 μM to 10 μM) or without treatment (mock). In all cases, cells were washed three times with acid solution (pH 3.5). After each treatment condition, an overlay of 1.5% carboxymethylcellulose (CMC) was added to the cells. Infected cells were incubated for 6 days at 37 °C and CO_2_ at 5% and subsequently fixed and stained with 2.5% crystal violet for plaque counting as described [63]. Plaque-forming units (PFU) were counted from three independent experiments.

### 4.6. Assessment of c-CrkII Phosphorylation by FCS

To evaluate the effect of imatinib on c-ABL kinase inhibition, c-CrkII phosphorylation in Tyr221 was measured. Briefly, Vero cells (2.5 × 10^4^ per glass coverslip) were treated with imatinib for 24 h, washed with PBS, fixed with 4% paraformaldehyde (PFA), and permeabilized with Triton X-100. Next, the cells were incubated with anti-phospho-Crk II and goat anti-rabbit Alexa fluor 594 (1:1000 dilution) for FCS. Measurements were carried out in a BD FACSCanto II (Becton Dickinson, Franklin Lakes, NJ, USA), and data were analyzed using FlowJo X software.

### 4.7. Assessment of c-ABL Silencing and c-CrkII Phosphorylation by ICWB

Vero cells were transfected with pFBAAVmU6miABL1-CMVeGFP SV40pA (amicABL) or pFBAAVmU6miScramble-CMVeGFPSV40pA (amiScr) in triplicate, and the expression of c-ABL and phospho c-CrkII was measured at 24, 36, 48, and 72 h post-transfection in 96-well culture plates by In-Cell Western (LI-COR Biosciences). Cells were fixed with 4% PFA, permeabilized with Triton X-100, and immunolabeled with anti-c-ABL or anti-phospho (Tyr221) Crk II, followed by anti-mouse or anti-rabbit antibodies labeled with IRDye 680LT (LI-COR Biosciences). Actin was used as loading control and detected with IRDye 800CW (LI-COR Biosciences). In addition, the expression of c-ABL and phospho c-CrkII was evaluated at 48 and 72 h post-transfection by FCS, as indicated in Section 4.6.

### 4.8. ENV Quantification

DENV envelope (ENV) protein was quantified at 1 h.p.i. and 22 h.p.i. by fluorescence microscopy (Zeiss Axio Observer Z1) and FCS. For ENV detection at 1 h.p.i., cells were transfected for 46 h, then infected with an MOI of 5 for 1 h at 4 °C and 1 h at 37 °C with 5% CO_2_. For detection at 22 h.p.i., cells were transfected for 24 h, then infected with MOI of 5 for 1 h at 4 °C and 22 h at 37 °C with 5 % CO_2_. At the end of the infection, Vero cells were washed twice with acid phosphate buffer (10 mM sodium hydrogen phosphate + 137 mM NaCl + 2 mM KCl, pH3.5) and twice with PBS, trypsinized, fixed with 4% PFA in cytoskeletal buffer, blocked with 3% Bovine Serum Albumin, permeabilized with Triton X-100, and immunolabeled with primary α-Envelope antibody (α-ENV) followed by goat-anti mouse AlexaFluor 594. FCS samples were evaluated as in Section 4.6, and microscopy fluorescence images were analyzed with Image J 1.53t software.

### 4.9. F-Actin Evaluation

F-actin reorganization was estimated at 1 h.p.i. and 22 h.p.i. by fluorescence microscopy (Carl Zeiss, Axio Observer Z1). For entry events (1 h.p.i.), cells were transfected for 46 h, then infected with an MOI of 5 for 1 h at 4 °C and 1 h at 37 °C with 5% CO_2_. For detection at 22 h.p.i., cells were transfected for 24 h, then infected with MOI 5 for 1 h at 4 °C and 22 h at 37 °C with 5% CO_2_. At the end of the infection, Vero cells were washed twice with PBS, trypsinized, fixed with 4% PFA in cytoskeletal buffer, blocked with 3% Bovine Serum Albumin, permeabilized with 0.1% Triton X-100, and immunolabeled with phalloidin AlexaFluor 594. FCS samples were evaluated as in Section 4.6, and microscopy fluorescence images were analyzed with Image J software. Actin stress fibers were quantified using a modification of the protocol we have described to analyze focal adhesions [64], whereas actin aggregates were quantified manually by a blind observer.

### 4.10. Statistics

Analyses include the mean and standard error of the mean (SEM) or standard deviation (SD) from three independent experiments. p-values were determined by two-tailed unpaired Student’s *t*-test (***, *p* < 0.001; **, 0.001 < *p* < 0.01; *, 0.01 < *p* < 0.05) using SPSS v.20 software and Graph Pad Prism 5.

## Data Availability

The original contributions presented in this study are included in the article. Further inquiries can be directed to the corresponding authors.
